# Are Complexity Metrics Reliable in Assessing HRV Control in Obese Patients During Sleep?

**DOI:** 10.1371/journal.pone.0124458

**Published:** 2015-04-20

**Authors:** Ramona Cabiddu, Renata Trimer, Audrey Borghi-Silva, Matteo Migliorini, Renata G. Mendes, Antonio D. Oliveira Jr., Fernando S. M. Costa, Anna M. Bianchi

**Affiliations:** 1 DEIB, Dipartimento di Elettronica, Informazione e Bioingegneria, Politecnico di Milano, Milano, Italy; 2 Cardiopulmonary Physiotherapy Laboratory, Federal University of São Carlos, São Carlos, São Paulo, Brazil; 3 Sleep Institute of São Carlos, São Paulo, Brazil; University of Modena and Reggio Emilia, ITALY

## Abstract

Obesity is associated with cardiovascular mortality. Linear methods, including time domain and frequency domain analysis, are normally applied on the heart rate variability (HRV) signal to investigate autonomic cardiovascular control, whose imbalance might promote cardiovascular disease in these patients. However, given the cardiac activity non-linearities, non-linear methods might provide better insight. HRV complexity was hereby analyzed during wakefulness and different sleep stages in healthy and obese subjects. Given the short duration of each sleep stage, complexity measures, normally extracted from long-period signals, needed be calculated on short-term signals. Sample entropy, Lempel-Ziv complexity and detrended fluctuation analysis were evaluated and results showed no significant differences among the values calculated over ten-minute signals and longer durations, confirming the reliability of such analysis when performed on short-term signals. Complexity parameters were extracted from ten-minute signal portions selected during wakefulness and different sleep stages on HRV signals obtained from eighteen obese patients and twenty controls. The obese group presented significantly reduced complexity during light and deep sleep, suggesting a deficiency in the control mechanisms integration during these sleep stages. To our knowledge, this study reports for the first time on how the HRV complexity changes in obesity during wakefulness and sleep. Further investigation is needed to quantify altered HRV impact on cardiovascular mortality in obesity.

## Introduction

Overweight and obesity represent a major public health concern. According to the World Health Organization, worldwide prevalence of overweight and obesity has almost doubled since 1980, with over one billion adults currently affected, eliciting concerns about the effects of increased adiposity on health [1]. It is well established that obesity is associated with increased all-cause mortality [2]. Specifically, obese patients have significantly increased death rates from cardiovascular (CV) disease [2, 3]. This might be explained by a number of factors; for instance, it has been hypothesized that obesity related dyslipidemias, in conjunction with chronic inflammation, may contribute to endothelial dysfunction and macrovascular changes, which might promote the development of CV disease [4]. Nonetheless, other factors might contribute to establishing a relationship between excess adiposity and increased mortality. Obesity was reported to be associated to an imbalance in the autonomous nervous system (ANS) activity, characterized by an increase in the parasympathetic tonus and by inappropriate activation of the sympathetic nervous system [5, 6]. Abnormal autonomic control of cardiac activity was documented to be associated with an increased risk for adverse CV events and mortality [7]. Autonomic functionality can be easily assessed by analyzing the heart rate variability (HRV), as the periodic fluctuations of the heart rate (HR) are associated with the relative contributions of the sympathetic and parasympathetic components of the ANS to the heart [8]. Reduction of HRV has been reported in several diseases and was shown to be an independent risk factor for the development of CV disease and for all-cause mortality [9]. Apart from autonomic anomalies, obese patients also present reduced sensitivity of the sino-atrial node to both sympathetic and vagal influences [10]. It is therefore likely that the adaptation of the HR to changing requirements is faulty in obesity. The CV system behavior is best characterized as a time-varying process exhibiting rhythmic and complex patterns [10]. Specifically, HRV has been recognized to follow a complex behavior, suggesting that it is controlled by complex chaotic neural, chemical and hormonal networks which are not strictly regular, but exhibit fluctuations across multiple time scales. Complexity is related to the amount of structured information contained in a signal; physiologic complexity theory suggests that healthy systems present higher complexity, i.e., nonlinear, fractal variability at multiple scales and long-range correlations [11]. A careful assessment of the HRV offers clues to this complexity and allows to investigate the adaptive capabilities and the stability of regulation of the underlying system, with higher complexity suggesting better adaptability and flexibility [11]. Alterations in the fractal and regularity structures of HRV can affect both short- and long-term mechanisms of complexity and could affect the system in terms of CV outcome and CV risk. Most studies on HRV investigate autonomic CV control by implementing classical linear methods, such as time domain and frequency domain analysis. [8]. However, in order to quantify different aspects of the CV control related to the system non-linear dynamics, an increasing interest is being focused on the study of the CV system complexity, which classical linear tools are not able to adequately assess [12]. In order to perform HRV complexity analysis, a number of entropy-derived nonlinear indices have been proposed, including approximate entropy and corrected conditional entropy [13, 14]. According to literature, disease is normally characterized by reduced complex variability in the temporal patterns of HR [15]. It has been shown that attenuation of HRV complexity characterizes, among others, CV and metabolic diseases [10].

In recent years, a growing interest has been manifested on autonomic cardiac modulation during sleep, deriving from the evidence that many sleep disorders, including insomnia and sleep disordered breathing, have been proved to be associated with CV dysfunction [16]. Sleep is a complex state characterized by important modifications of the CV autonomic regulation: during deep sleep the vagal drive prevails, while rapid eye movement (REM) phases exhibit increased sympathetic modulation and reduced parasympathetic control [17]. The application of non-linear methodologies to HRV investigation during sleep revealed that different sleep stages present distinctive non-linear properties. For instance, non-REM (NREM) sleep was found to be characterized by increased sample entropy [18] and a reduced detrended fluctuation analysis exponent [19]. As for REM sleep, evidence confirmed it to be characterized by non-linear properties, but results are controversial [12].

To our knowledge, no studies have reported on how the HRV complexity changes in obesity across different sleep stages and wakefulness. In order to do so, as each sleep stage lasts for generally reduced portions of time, complexity measures need be calculated on short-term signals, with duration of around 10 minutes. Such application is not common, as results presented in literature related to complexity measures are normally derived on long-period signals, with duration of several hours [20]. Nonetheless, recent developments have shown various measures of complexity to be suitable to quantify complexity over short data sequences (∼300 samples) [21, 22, 23]. A preliminary analysis was therefore performed in order to evaluate the reliability of a set of complexity measures when applied on short-period signals. Experimental data coming from healthy and obese subjects were then analyzed with the purpose to investigate HRV complexity by means of a set of non-linear indices during wakefulness and sleep, in order to assess anomalies in the pathological condition with respect to the physiological state.

## Materials and Methods

### Subjects

Eighteen obese patients who were referred to the Sleep Institute of São Carlos, São Paulo, Brazil, for evaluation of excessive daytime sleepiness prior to bariatric surgery were involved in the study. Twenty eutrophic subjects were included in the study as comparison cohort. Participants’ demographic characteristics are reported in Table 1.

**Table 1:** Demographic, clinical and biochemical characteristics in healthy controls and obese patients.

	Control subjects (n = 20)	Obese patients (n = 18)
**Age (yrs)**	45.2 ± 8.0	49.9 ± 11.0
**Male**	5	9
**Female**	15	9
**BMI (kg/m** ^**2**^ **)**	25.5 ± 1.7	42.7 ± 9.2*
**AHI (h** ^**-1**^ **)**	2.2 ± 1.7	43.8 ± 23.4*
**Basal Saturation %**	95.40 ± 1.58	94.12 ± 3.64
**Mean Saturation %**	93.30 ± 0.95	88.18 ± 7.20*
**T90 (min)**	1.6 ± 2.8	117.7 ± 102.5*
**ODI (h** ^**-1**^ **)**	2.4 ± 2.7	37.4 ± 21.8*

Data are presented as mean ± SD.

* denotes significant difference (p ≤ 0.05) when compared with control group. BMI: Body Mass Index; AHI: Apnea Hypopnea Index; T90: time spent below 90% oxygen saturation; ODI: Oxygen Desaturation Index.

### Data collection

The study protocol was approved by the Ethics Committee of Federal University of São Carlos (CEP-UFSCar: opinion N. 401/2010). All participants provided their written informed consent to participate in the study. Prior to nocturnal in-laboratory examination, all subjects completed a validated self-administered questionnaire concerning possible daily or nocturnal symptoms, intoxications, medication and medical history. None of the patients were on beta-blockers, nor was any of them using drugs which could affect the ANS functionality. All participants underwent general examination. For each subject the body mass index (BMI), a good measure of general adiposity, was calculated as the weight [kg] divided by the square of the height [m].

All participants underwent an overnight comprehensive sleep study performed through polysomnography (PSG). By using Icelera Fast-Poli 26i device (*Homed*, *São Paulo*, *Brazil*), the electrocardiogram (ECG) was acquired for each subject, with a sampling frequency of 256 Hz. Electroencephalogram (EEG), electro-oculogram (EOG), oronasal flow, thoracoabdominal movement, snoring and body position were concurrently acquired for each subject. The sleep architecture was derived by an expert physician through visual scoring of the EEG, the EOG and the EMG; wakefulness, sleep stages N1, N2 and N3 and REM phases were scored according to the standards recommended in the American Academy of Sleep Medicine (AASM) Manual for the Scoring of Sleep and Associated Events [24]. Apnea events were concurrently detected by visual examination of the thoracic and abdominal respiration signals, of the nasal airflow and of the snoring intensity. For each subject the Apnea Hypopnea Index (AHI), a commonly used descriptor of Obstructive Sleep Apnea (OSA) severity, was calculated as the number of apneic and hypopneic events per hour of sleep [25].

Basal saturation %, defined as the saturation measured during wakefulness, mean saturation %, time spent with oxygen saturation lower than 90% (T90) and the oxygen desaturation Index (ODI), defined as the number of desaturation events per hour of sleep, were derived from the PSG recordings.

### Signal processing

The RR-interval signals were extracted from each ECG signal by implementing the Pan-Tompkins algorithm [26]. Parabolic interpolation of QRS was used to refine the R peak detection. In order to identify and eliminate outliers due to artifacts present in the original ECG signal, attributable to mechanical noise during the signal acquisition, the obtained RR sequence was filtered using a 1000 sample window moving average filter and samples were considered as outliers when the following condition was met:

$$\left|{\text{RR}}_{\text{i }}-{\text{ AVE}}_{100}\right|>5{\text{*SD}}_{100}$$ (1)

where RR_i_ is the i^th^ sample in the tachogram and AVE_100_ and SD_100_ are the average and the standard deviation values of the previous 100 samples without outliers, respectively. Identified outliers were then replaced with the value of the corresponding sample in the filtered RR sequence. Each RR series was analyzed to identify 10 minute signal portions within the subject’s wakefulness period, deep sleep stages N2 and N3 and REM phase. Sleep stage N1 signal portions were not considered in the present study as their duration and stationarity were not judged sufficient to perform the analysis. In order to minimize circadian rhythm effects and differences attributable to the physiological sleep cycle succession, signal portions were always selected during the central part of the night (third or fourth sleep cycle, depending on each patient’s signal). Signal processing was performed in the MATLAB environment, version R2012a, (*The Mathworks Inc*., *Natick*, *MA*).

### Analysis validity

In order to confirm the validity of the analysis which would be subsequently performed over the extracted 10 minute signal portions, a reliability analysis was conducted. Since a set of non-linear complexity measures (which will be presented in the following sections), normally used on long-period signals [20], would be performed on short-time signal portions, the authors used such short-time signal portions to simulate long-time signals. Only RR sequences obtained from control patients were used to perform the simulation. Complexity measures were then calculated on simulated long-time and short-time signals with different lengths and results were compared to evaluate the actual possibility of using such non-linear measures to characterize short-time signals. For each sleep stage and for the wakefulness state the tachogram of each healthy subject was processed in order to model it using an autoregressive (AR) model, whose general expression is as follows:

$$y(t)=\sum_{k=1}^{p}a_{k}y(t-k)+\varepsilon (t)$$ (2)

where *y(t)* is the series under investigation, *a*_*k*_ are the autoregressive coefficients and *ε(t)* is the residual term, i.e. a white noise. The model order was chosen using the Akaike Information Criterion (AIC) and the model coefficients were obtained using the least squares method based upon the Yule-Walker equations [27]. The estimated parameters and the white noise variance were used to simulate 10 hour long signal portions. A set of non-linear complexity measures (which will be presented in the following sections) were derived for each 10 hour long simulated signal and the average value was calculated over all signals. Each simulated signal was subsequently divided into smaller portions, corresponding to 5 hour, 2 hour, 1 hour, 30 minute and 10 minute time epochs. For each time duration, non-linear measures were derived on the obtained signal portions and average values were calculated over all epochs corresponding to each time duration. A comparison was conducted among results obtained for different time epochs, with the objective to assess the robustness of each non-linear complexity measure with changing time duration, and, specifically, to determine whether each measure is still reliable when calculated over shorter periods of time. The parameters that were considered are defined in the following sections. The analysis validity was performed in the MATLAB environment, version R2012a, (*The Mathworks Inc*., *Natick*, *MA*).

### Non-linear complexity measures

#### Sample entropy (SE)

SE is a well validated complexity measure obtained by comparing a time series to a given pattern of length *m* and gives an indication about its regularity, with a tolerance r [14]. *m* represents the detail level at which the signal is analyzed and *r* is a threshold used to identify irregularities. In the present study *m* = 2 and *r* = 0.2 were adopted, on the basis of previous works [20].

#### Lempel-Ziv complexity (LZC)

The LZC algorithm is used to characterize the randomness of finite sequences [28]. This index gives a measure of the algorithmic complexity, which refers to the irregularity of a dynamic process. For a random signal, the algorithmic complexity corresponds to the length of the signal, indicating that any compression attempt will result in information loss. The LZC algorithm has been applied to various biomedical signals, including ECG, EEG and EMG signals, in order to determine the signal level of complexity. In order to apply the LZC algorithm to a biological signal, this needs to be transformed into a quantized sequence by comparison with one or more thresholds. By using *n* thresholds, the signal will be quantized into *n+1* levels. Depending on the number of levels, different indices can be calculated. In the present work two indices, LZC 1 and LZC 2, were computed, obtained by quantizing the signals to 2 and 3 levels, respectively. To compute LZC 1, the threshold was chosen as the signal 50^th^ percentile. To compute LZC 2, the thresholds were chosen as the signal 33^rd^ and 66^th^ percentiles. The LZC algorithm is thoroughly described elsewhere [28].

#### Detrended fluctuation analysis (DFA)

The DFA is a method that allows to quantify correlation properties in stationary and non-stationary time series of biological origin [29]. DFA advantages over conventional methods include the capability of detecting intrinsic self-similarity in seemingly non-stationary time series and the possibility of avoiding the detection of apparent self-similarity, which may be nothing more than artifacts [30]. First introduced by Peng et al., DFA has been widely applied to studies of correlations in HRV signals and has proven useful in discriminating between physiological and pathological conditions [31].

Long-range and short-range correlation properties can be quantified by taking into account different time scales. The algorithm, which is thoroughly described elsewhere [20], allows to characterize the relationship between the mean time series fluctuation *F(n)* and the considered scaling exponent *n* (corresponding to the number of samples considered for each computation). *F(n)* usually increases as the scaling exponent *n* becomes larger. A linear relationship on a log-log plot is found when power law scaling is present. The fluctuations can therefore be characterized by a scaling exponent, which is the slope of the line relating *log F(n)* to *log n*. Uncorrelated fluctuations lead to DFA = 0.5, while DFA > 0.5 indicates positive temporal correlations and DFA < 0.5 indicates anti-correlations. In the present work a long-term exponent for 100 ≤ *n* ≤ 1000 was estimated.

### Analysis

The average values of each non-linear measure were calculated for the normal population and for the obese patients on the experimental 10 minutes long signal portions during wakefulness and sleep stages N2, N3 and REM, in order to comprehensively assess the HRV physiologic complexity in our study sample. The analysis was performed in the MATLAB environment, version R2012a, (*The Mathworks Inc*., *Natick*, *MA*).

### Statistical analysis

An *a posteriori* power analysis was performed using the G*Power statistical package, version 3.1.9.2 (*F*. *Faul*, *Universität Kiel*, *Germany*), to calculate our results statistical power. Considering our study total sample size of 38 subjects and a 5% error, statistical power was calculated to be 99%.

An ANOVA (ANalysis Of VAriance) analysis, with Dunn’s *post hoc* test, was conducted on results obtained from the preliminary analysis with the objective to assess, for each non-linear complexity measure, any significant differences (p-value ≤ 0.05) between the parameters evaluated for different time duration signals and, specifically, to determine whether each measure is still reliable when calculated over shorter periods of time. As for the actual analysis conducted on the experimental data, an ANOVA analysis, with Dunn’s *post hoc* test, was performed. *Post hoc* tests were performed between groups and within groups between each pair of conditions (wakefulness, N2, N3 and REM). A correlation analysis was conducted for each population between each parameter, evaluated during wakefulness and sleep, and clinical parameters. Statistical analysis was performed in the MATLAB environment, version R2012a, (*The Mathworks Inc*., *Natick*, *MA*).

## Results

The participants’ demographic, clinical and biochemical characteristics are summarized in Table 1.

According to the BMI grading system described by Ravesloot *et al*.[32], five patients were classified as mildly obese (BMI 30–34.9 kg/m^2^), one patient was classified as severely obese (BMI 35–39.9 kg/m^2^), seven patients were classified as morbidly obese (BMI 40–49.9 kg/m^2^) and five patients were classified as super obese (BMI > 50 kg/m^2^).

According to the AHI grading system described by Ruehland *et al*. [33], six patients were classified as affected by moderate OSA (AHI 15-30/hour) and twelve patients were classified as affected by severe OSA (AHI > 30/hour).

Significantly different BMI, AHI, mean saturation %, T90 and ODI values (p-value ≤ 0.05) were found in the obese patients (BMI = 42.7 ± 9.2 kg/m^2^; AHI = 43.8 ± 23.4 h^-1^; mean saturation % = 88.18 ± 7.20; T90 = 117.7 ± 102.5 min; ODI = 37.4 ± 21.8 h^-1^) when compared to controls (BMI = 25.5 ± 1.7 kg/m2; AHI = 2.2 ± 1.7 h^-1^; mean saturation % = 93.30 ± 0.95; T90 = 1.6 ± 2.8 min; ODI = 2.4 ± 2.7 h^-1^). In the obese population, ODI positively correlated with BMI (r = 0.58) and with AHI (r = 0.78). In the control group BMI positively correlated with AHI (r = 0.78), with T90 (r = 0.78) and with ODI (r = 0.82) and negatively correlated with mean saturation % (r = -0.75); AHI positively correlated with T90 (r = 0.87) and with ODI (r = 0.96) and negatively correlated with mean saturation % (r = -0.82).

**Fig 1:**
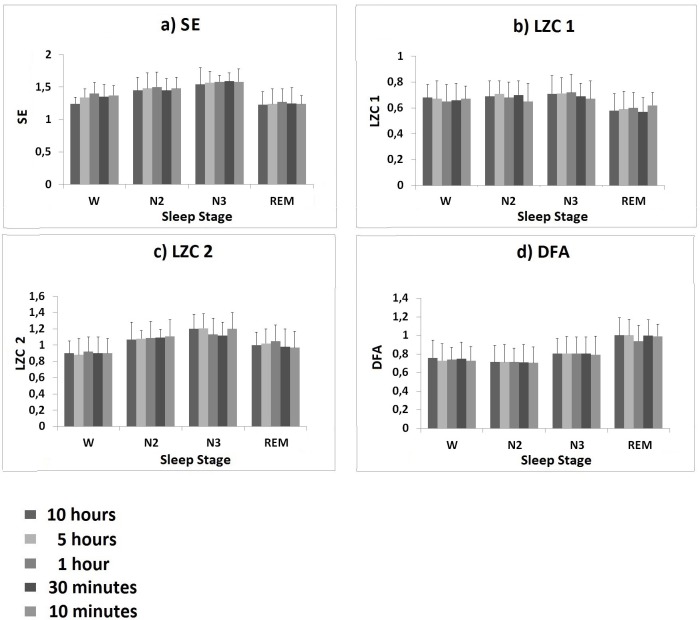
Results of the preliminary analysis performed to evaluate the reliability of complexity measures when applied on short-period signals. a) SE, b) LZC 1, c) LZC 2 and d) DFA mean values (± SD) calculated on 10 hour, 5 hour, 1 hour, 30 minute and 10 minute long simulated signal portions, during wakefulness and sleep stages N2, N3 and REM.

Results of the preliminary analysis aimed at evaluating the reliability of our set of complexity measures when applied on short-period signals are reported in Fig 1.

All parameters changed in the passage from wakefulness to deep sleep to REM sleep following a similar pattern for different duration signals; specifically, SE, LZC 1 and LZC 2 increased during deep sleep stage N3, with respect to wakefulness and decreased during REM sleep. As for DFA, values increased during REM sleep with respect to wakefulness and other sleep stages. However, for all complexity measures, the ANOVA analysis did not show any significant differences among the parameter evaluated for different time durations.

On the basis of the evidence of suitability to reliably characterize short-term signals, all four parameters were taken into account in the subsequent analysis performed on our experimental data.

**Fig 2:**
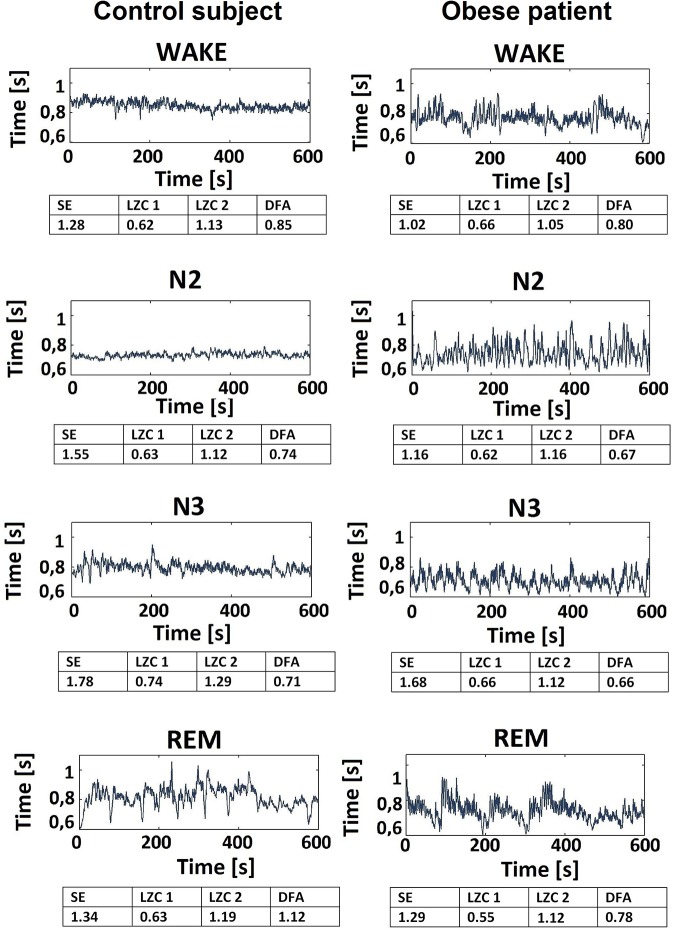
Typical example of tachogram signals for a healthy subject and for an obese subject. Tachogram during wakefulness and sleep stages N2, N3 and REM for a healthy subject (first column) and for an obese patient (second column). SE, LZC 1, LZC 2 and DFA values are reported for each signal portion.

The tachogram was studied for each subject. Typical examples of the signal for a healthy subject and for a patient are shown in Fig 2.

At a first glance, the healthy subject’s tachogram appears more regular during wakefulness and sleep stages N2 and N3 and less regular during REM sleep. The same behavior is less visible in the obese patient’s tachogram.

**Fig 3:**
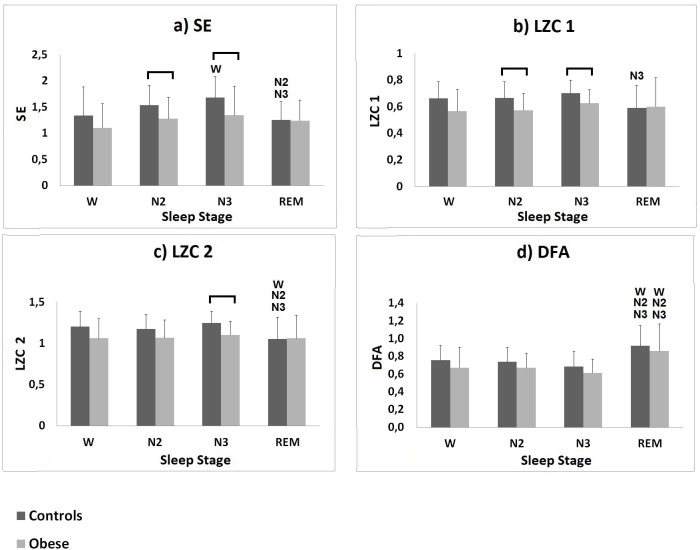
Results of the complexity analysis performed on the control group and on the obese group. a) SE, b) LZC 1, c) LZC 2 and d) DFA mean values (± SD) calculated for the controls (darker bars) and for the obese group (lighter bars) during wakefulness and sleep stages N2, N3 and REM. W denotes significant difference (p-value ≤ 0.05) when compared to wakefulness; N2 denotes significant difference (p-value ≤ 0.05) when compared to N2; N3 denotes significant difference (p-value ≤ 0.05) when compared to N3; the horizontal bar denotes significant difference between the two populations.

A set of non-linear parameters were calculated from the tachogram, for wakefulness and different sleep stages, according to the clinical classification summarized in the hypnogram. The average values of the evaluated HRV non-linear parameters during wakefulness and sleep stages N2, N3 and REM, for the obese and the healthy groups, are shown in Fig 3.

For both groups SE, LZC 1 and LZC 2 mostly increased during deep sleep stage N3 (obese group: SE = 1.34 ± 0.56; LZC 1 = 0.63 ± 0.10; LZC 2 = 1.10 ± 0.17; controls group: SE = 1.68 ± 0.40; LZC 1 = 0.70 ± 0.10; LZC 2 = 1.25 ± 0.14;), with respect to wakefulness (obese group: SE = 1.10 ± 0.46; LZC 1 = 0.57 ± 0.16; LZC 2 = 1.06 ± 0.24; controls group: SE = 1.34 ± 0.54; LZC 1 = 0.66 ± 0.12; LZC 2 = 1.20 ± 0.19) and sleep stage N2 (obese group: SE = 1.27 ± 0.41; LZC1 = 0.57 ± 0.13; LZC2 = 1.07 ± 0.22; controls group: SE = 1.54 ± 0.38; LZC1 = 0.67 ± 0.12; LZC2 = 1.17 ± 0.18); SE, LZC 1 and LZC 2 decreased during REM sleep (obese group: SE = 1.24 ± 0.39; LZC 1 = 0.60 ± 0.22; LZC 2 = 1.06 ± 0.28; controls group: SE = 1.26 ± 0.35; LZC 1 = 0.59 ± 0.17; LZC 2 = 1.05 ± 0.26;), reaching values comparable with those observed during wakefulness. A significant difference (p ≤ 0.05) was found in the control group for SE values between N3 and wakefulness, between REM and N2 and between REM and N3; for LZC1 values between REM and N3; for LZC2 values between REM and wakefulness, between REM and N2 and between REM and N3. All three parameters were found to be lower for the obese population during wakefulness and all sleep stages; a significant difference (p ≤ 0.05) was found between the two groups during sleep stage N3 for SE, LZC1 and LZC2 and during sleep stage N2 for SE and LZC1. It is interesting to observe that this is in contrast with what can be visually observed in Fig 2: at a first glance, the healthy subject’s tachogram appears more regular during wakefulness and deep sleep than the patient’s one; however, complexity metrics show that this is not the case.

For both populations DFA increased during REM sleep (obese group: DFA = 0.86 ± 0.30; controls group: DFA = 0.92 ± 0.23) with respect to wakefulness (obese group: DFA = 0.67 ± 0.23; controls group: DFA = 0.76 ± 0.16), to sleep stage N2 (obese group: DFA = 0.67 ± 0.17; controls group: DFA = 0.74 ± 0.16) and to deep sleep stage N3 (obese group: DFA = 0.62 ± 0.16; controls group: DFA = 0.69 ± 0.17). For both groups, a significant difference (p ≤ 0.05) was found for DFA values between REM and each of the other conditions.

**Fig 4:**
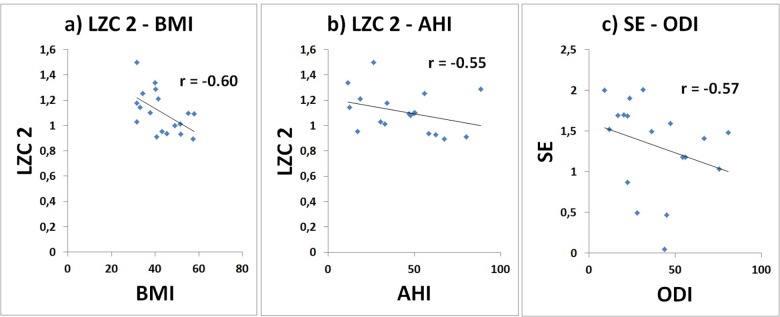
Significant results of the correlation analysis performed on complexity measures and clinical parameters. Correlation between a) LZC 2 and BMI, b) LZC 2 and AHI, and c) SE and ODI calculated for the obese population, during sleep stage N3.

A correlation analysis was conducted between each parameter, evaluated during wakefulness and sleep, and clinical parameters. Fig 4 shows that significant, moderate correlations were found for the obese population during sleep stage N3. LZC2 was negatively correlated with BMI (r = -0.60) and with AHI (r = -0.55); SE was negatively correlated with ODI (r = -0.57).

## Discussion

The current demographic trends and continued progression of obesity are leading health professionals and researchers to give increasing attention to the dangers of obesity [1]. Even though hospital admission rates for obesity have drastically increased over the last decades, inpatient care is often directed at dealing with associated conditions rather than obesity itself [34].

Obesity was shown to be accompanied by altered CV dynamics, which are associated with increased mortality risk [2, 3]. Unbalances in cardiac autonomic modulation have also been observed in obese patients [5, 6], which can be expected to adversely affect the CV prognosis [7]. Alterations in the autonomic function are reflected in the HRV signal [8]. Specifically, since the CV system is regulated by highly complex, non-linear mechanisms [35], by analyzing the HRV complexity it is possible to investigate the adaptive capabilities and the stability of regulation of the underlying regulation system [11].

Several approaches have been described in literature that were developed to capture the amount of structured information contained in a biological signal and, thus, to characterize a phenomenon complexity [13, 14].

In the present work the dynamics of HRV complexity were investigated during wakefulness and across different sleep stages in healthy and obese subjects by calculating a set of non-linear HRV indices, which included SE, LZC 1, LZC 2 and DFA. As each sleep stage lasts for reduced portions of time, complexity measures could only be calculated on short-term signals (∼ 10 minute long). This is not common, as non-linear complexity measures are normally used to characterize long-period signals [20]. Therefore, a preliminary analysis was performed aimed at verifying the reliability of the selected complexity measures when applied on short-term signals. For all parameters, results showed no significant differences in the values calculated over 10 minute long epochs (experimental signal portions) with respect to all other durations (simulated signal portions), for wakefulness and all sleep stages, thus confirming the possibility of using these parameters on short-term signals. In accordance with these results, all four parameters were taken into account in the subsequent analysis performed on our experimental data.

By looking at Fig 3, it is possible to infer that the investigated non-linear indices varied during sleep following a somewhat similar pattern in both groups, but being attested for the obese population around lower values than those observed for the control group (with the exception of SE, LZC 1 and LZC 2 during REM sleep, when comparable values were observed for the two groups).

SE changed as the subjects fell asleep and entered sleep stages N2, N3 and REM, following a similar pattern for the two populations. Specifically, SE increased during deep sleep, and decreased during REM sleep, in line with previous evidence [18]. A similar pattern was followed by LZC 1 and LZC 2. The lower SE and LZC values observed in the obese population can be indicative of a shift of the sympatho-vagal balance toward the sympathetic component [13]. Significantly lower complexity was observed in the obese population during sleep stages N2 (for SE and LZC 1) and N3 (for SE, LZC 1 and LZC 2). This is consistent with the concept of physiologic complexity, according to which the complexity characterizing the human subsystems in physiological conditions decreases with aging and illness [36]. Generally, a decrease of complexity is indicative of a CV control simplification [12]. In physiological conditions, CV regulation results from the interaction among different control systems; in pathological conditions, some of these mechanisms prevail, with the others being inhibited or impaired; this results in a simplification of the CV control which, in the long term, can reduce the CV system flexibility and increase its susceptibility to adverse conditions [12]. Our results, therefore, suggest that obesity is associated with a significant decline of complexity in CV control during NREM sleep. This complexity decrease could reflect a potential impairment in the CV system capability to respond to perturbations and to adapt to changing conditions during NREM sleep, which in turn might determine a state of increased CV risk [12], especially when associated to major CV diseases, by which obese patients are often affected [2, 12].

Long-range correlations have been observed in heartbeat dynamics for a long time [37] and have been found to be affected by pathological conditions, autonomic functionality and circadian rhythm [31]. In the present work, the presence of long-range correlations (DFA >> 0.5) was observed in both groups during wakefulness and sleep. For both groups, the ANOVA revealed a significant difference (p ≤ 0.05) between REM and all other conditions, with the highest DFA value during REM sleep, suggesting that long-term correlations were stronger during REM sleep and much weaker during wakefulness and NREM sleep. This finding, consistent with previous results, indicates an enhanced control on autonomic functions by higher brain structures during REM sleep [31]. The weaker correlations observed for both groups during NREM deep sleep (DFA ≈ 0.6) are indicative of a somewhat random regulation of the heartbeat during this sleep phase and suggest a weaker involvement of higher brain regions in the autonomic regulation of cardiac activity [38]. This interpretation is in accordance with previous studies which revealed the presence of long-range correlations in the fluctuations of brain wave amplitudes and frequencies during REM sleep, but not during deep sleep [39].

Taken together, our results showed that obese patients exhibited significant reductions in HRV complexity during NREM sleep as compared with controls. This reduction might be attributed to a breakdown of the underlying regulatory mechanisms, but questions remain about the origin of HRV complexity and the mechanisms involved in its reduction in pathological conditions [15]. The CV system is regulated by complex neural mechanisms which rely on the interaction among various subsystems [35]. The overall CV variability is also modulated by external noise and state changes, to which, in physiological conditions, the CV system adapts. The significant loss of HRV complexity we observed in the obese population during NREM sleep suggests that CV regulation may have a reduced ability to control its different underlying subsystems during this phase [12]. As a result, this might lead to a deficiency in the integration of control mechanisms, which in turn limits the ability of the system to react to perturbations, thus increasing these patients’ risk for CV adverse events [40].

It is important to point out that the study presents some limitations, including no matching gender controls for each pathological subject. Moreover, the effects of the intrinsic autonomic control characterizing sleep cycles and stages progression and those of respiration were not taken into account. As for the subjects’ respiration, HRV modifications due to the presence of OSA [41], specifically in the obese population, should be considered in future studies. A strong association between OSA and the risk for CV events has been reported [42]. Recently published studies [42, 43] showed that treatment with continuous positive airway pressure (CPAP) devices is not only able to improve signs and symptoms of OSA in controls and obese patients, but leads to CV outcome improvements and beneficial effects on CV risk factors. Such recent developments, based on linear measurements of HRV, suggest that CPAP can reverse some of the HRV changes, thus indicating that the damage could be not permanent. Further studies should be performed to analyze CPAP treatment effects on non-linear HRV indices and to see if the alterations we observed are reversible by using CPAP.

Correlation properties between non-linear parameters and clinical data during sleep in obese patients should also be investigated in better detail, with specific focus on differences across sleep stages. Our results suggest increased correlation between the investigated parameters and clinical data during deep sleep, but more comprehensive results are needed to formulate a hypothesis as to why this occurs.

Summing up, the present study confirmed SE, LZC 1, LZC 2 and DFA as reliable measures of complexity even when used to characterize short-term signals. Our findings support a central role of excessive adiposity in influencing CV variability during sleep. In accordance with the theory that affirms that complexity reflects the capability of the CV system to respond to perturbations [11], reduced HRV complexity which accompanies obesity during sleep might represent an index of increased CV risk in these patients [12]. However, further investigation is needed in order to establish whether HRV complexity alterations may actually impact on CV morbidity or mortality and to evaluate the CV risk during specific sleep stages in obese subjects; this, together with the identification of potential sleep-related high risk moments, might lead to a novel, effective approach for prevention of cardiac major events in obese patients during sleep.

## Supporting Information

S1 Dataset Controls’ data analyzed in the present study. (ZIP)

S2 Dataset Patients’ data analyzed in the present study. (ZIP)
